# The effects of Chinese proprietary medicine and vaccination on patients with COVID-19: a retrospective study in Macao

**DOI:** 10.1186/s13020-023-00877-8

**Published:** 2024-01-23

**Authors:** Hui Mo, Man-Fei Zhou, Edmundo Patricio Lopes Lao, Ka-Kei Chan, On-Na Lai, Man-In Ho, Kin-Wa Wong, Ka-Meng Ho, Kin-Tim Sio, Keng-Lam Fong, Yong-Hua Zhao, Seng-Ip Cheang, Iek-Long Lo

**Affiliations:** 1 Government of Macau SAR-Health Bureau, Edifício da Administração dos Serviços de Saúde, Rua Nova à Guia, no. 39, Macao SAR, 999078 China; 2 State Key Laboratory of Quality Research in Chinese Medicine, Institute of Chinese Medical Sciences, University of Macau, Taipa, Macao SAR, 999078 China; 3 Chinese Medicine Anti-Epidemic Team of the Health Bureau, Macao SAR, 999078 China

**Keywords:** COVID-19, Chinese proprietary medicine, Lianhua Qingwen capsule, Huoxiang Zhengqi capsule

## Abstract

**Background:**

COVID-19 is continuing to ravage globally and has resulted in a huge health and financial burden. Chinese proprietary medicines, such as Lianhua Qingwen (LHQW) and Huoxiang Zhengqi (HXZQ) capsules, have been recommended for non-high-risk patients with COVID-19 in China. Based on this, we described the baseline information, using status of LHQW and HXZQ capsules and inoculation history of quarantined patients in the second half of 2022 in Macao. Additionally, we analyzed the underlying association among medicines administration, vaccination and COVID-19 indices, in order to explore novel clues for the regular control and prevention of local epidemic situation in the future.

**Methods:**

A total of 976 patients in Macao quarantine hotels from June to August 2022 were included in the present study, of which, 857 subjects were followed-up for prognosis evaluation. During quarantine, the baseline demographic information, including sex, age, BMI, occupation and personal habits were collected. Additionally, the inoculation history, medicine employment status and cycle threshold (Ct) values were also reported. We interviewed the patients for collection of their symptoms at the beginning and end of quarantine, as well as prognostic ones. Basic statistical description of baseline information, vaccination history and medication were displayed. Chi-squared test or with continuous correction test was employed for comparison of dichotomous data between two or multiple groups. Binary logistic regression was applied to reveal the correlation between potential risk factors and Ct values or prognosis symptoms. We also used Cox regression model to identify the effect of different types of vaccine products on Ct value altering rate.

**Results:**

Patients who were female (52.0%), engaged in service industry (31.8%), from Macao native (65.8%), never took physical exercises (33.6%) and preferred irritated diet (59.5%) enjoyed more dominant proportions. Over 80% of participants were inoculated and 74.6% of them chose inactivated COVID-19 vaccine produced by China National Biotech Group (CNBG). Participants used LHQW capsules accounted for 92.1% and the duration of medicating lasted for one to two weeks. All of the reported symptoms were significantly ameliorated after quarantine and the duration of quarantine was concentrated on 21 days. People with different age, sex, occupation and region had different choices of HXZQ administration and vaccination. Additionally, middle dose (4–5 boxes) of LHQW capsules exhibited evidently negative association with positive Ct values (adjusted, − 0.037 ± 0.19, *p* = 0.04). Two doses of CNBG and one dose of mRNA vaccine had obvious protective effect on reducing Ct positive rate (*p* = 0.041). Meanwhile, symptoms after quarantine were significantly positive correlated with those in prognosis (adjusted, 1.38 ± 0.18, *p* < 0.0001).

**Conclusion:**

Our study found that the administration of LHQW capsules was beneficial for Ct value turning negative, meanwhile, certain mixed inoculation may be the promoting factor to reduce the positive rate of Ct value. These findings provide data basis for the Chinese proprietary medicine treatment and mixed vaccination applying for prevention and control of local COVID-19 epidemic in the future.

**Supplementary Information:**

The online version contains supplementary material available at 10.1186/s13020-023-00877-8.

## Introduction

2377 confirmed cases were in Macao by the end of 2022 due to the pandemic of COVID-19, accounting for 0.35% of local total population [1]. High dense population in small area is the major feature of Macao demography, a total population of over 680,000 is counted on the area of 32.9 square kilometers (density of over 20,000 people per kilometer) [2], which is extremely higher than that of Chinese mainland, easily bringing convenience for the COVID-19 virus effective dissemination [3]. In addition, as a well-known tourist and harbor region, considerable short-term population mobility is typical here. According to the statistical yearbook of Macao in year 2022, apart from the over 80% of tourists from Chinese mainland, ones from Hongkong, Taiwan and abroad make up of the rest parts of visitors, and their average sojourn time is usually less than one week [4]. Although Macao government restricted entry and exit at the border during the outbreak and kept strict records of the fever status of each person, the daily basic population motility and import/export trade continued to pose a challenge to the tracking and control of the epidemic [5].

As a heavily mutated COVID-19 strain, Omicron variant had spread worldwide and led to severe acute respiratory syndrome for infected patients [6]. Different from the epidemic strain in Chinese mainland, the main subvariant of Omicron raged locally was BA.5.1 in Macao, which originated directly from the evolutionary branch of Omicron BA.2. Owing to the higher transmission rate than other subvariants, BA.5.1 infected a large number of populations in a quite short time, inducing systemic and upper respiratory symptoms, such as fever, runny nose, sore throat, fatigue and so on [7]. Thus, the wide employment of antiviral drugs sprung up according to the guidelines issued by National Health Commission of the People’s Republic of China and World Health Organization (WHO) [8, 9]. However, there remains limitations on these drugs treatment. For example, the adverse events, such as diarrhea, nausea and dizziness were non-negligible [10, 11]. In the safety research on Molnupiravir and Nirmatrelvir-Ritonavir, it showed that the above adverse reactions accounted for 1% to 2% of participates in the treatment group [12]. Additionally, it was unknown whether these drugs still had ideal efficacy against the novel mutations in the future [13].

The investigation suggested that 90% of Chinese patients infected by COVID-19 received Chinese medicines treatment [14]. Recent study demonstrated Chinese medicines could decrease the proportion of severe case progressing by 55%, as well as the mortality rate of severe or critical patients by 49% [15]. Among these medicines, early in 2020, Lianhua Qingwen (LHQW) capsule was designated as one of the commonly recommended drugs against COVID-19 by National Health Commission of the People’s Republic of China [16]. In fact, there were series of Chinese proprietary medicines employed during pandemic, and most of them were the symptomatic treatment [14]. For example, LHQW capsule was mainly applied for the symptoms of sore throat, fever, and muscle soreness, while another common medicine, Huoxiang Zhengqi (HXZQ) capsule was used to alleviate asthenia with gastrointestinal discomfort, such as vomiting and diarrhea.

Apart from the Chinese medicine treatment, due to the wide variability and the strong immune escape ability, the infected patients had a higher risk of re-infection [17]. Hence, the vaccination is an essential option to reduce the corresponding health risks. Updated to the end of 2022, over one million times of vaccination were registered, and over six hundred thousand people were vaccinated [1]. One report from the United States suggested that authorized mRNA vaccines were highly effective among working-age adults on preventing COVID-19 infection, which ameliorated the viral RNA load, fever symptoms and the duration of illness [18]. Meanwhile, according to domestic research, inoculation of COVID-19 inactivated vaccine had a significant effect on attenuating the severity rate of elderly people over 60 years old [19]. However, the exact statistical reports of Chinese proprietary medicine treatment and vaccination in Macao are still lacking. Therefore, we retrospected the situation of a group of subjects who were quarantined in local hotels due to COVID-19 infection from June to August 2022, and collected their baseline information, including Chinese proprietary medicine treatment and vaccination history. Besides, we also recorded their cycle threshold (Ct) values, as well as symptoms from the beginning of quarantine and followed up after they left the quarantine environment. Patients took Chinese proprietary medicine under the guidance of Chinese Medicine doctors, and all medication information was tracked and uploaded by qualified personnel. In the present study, we mainly discussed two kinds of common Chinese proprietary medicines, the effects of LHQW and HXZQ capsules on COVID-19 infected patients, along with the vaccination, in order to provide a factual and statistical basis for the future epidemic control and therapeutic policies in Macao.

## Methods

### Subjects enrollment

Participants in the present study were recruited from June 18th to August 26th, 2022, in Macao quarantine hotels, including Sheraton Hotel, England Hotel and England Marina Club Hotel. The inclusion criteria were listed as below: (1) agreed to receive this interview; (2) did not take any western antiviral drugs, but only used designated Chinese proprietary medicine under the guidance of Chinese Medicine doctors. Meanwhile, patients who met the following conditions were excluded: (1) declined the present survey; (2) failed to understand the meaning of questions during the interview or had communication barriers due to language or hearing problems; (3) had cognitive impairment or suffered from any disease that might lead to cognitive disorders; (4) severe cardiovascular, cerebrovascular, or other chronic diseases; (5) had other severe infectious diseases. Taken together, 976 subjects were included in the survey and during the period after the end of quarantine, a total of 857 patients participated in the follow-up survey via telephone interview. Since this study is conducted based on telephone interviews, written consents were waived but verbal informed consents were obtained from all participants or their legal guardians prior to the survey.

### Reagents and drugs

LHQW capsules were obtained from Yiling Pharmaceutical by Health Bureau of the Government of the Macao Special Administrative Region. HXZQ capsules (Shenwei Pharmaceutical) were provided by the State Administration of Traditional Chinese Medicine (TCM).

### Study variables

According to current studies on COVID-19 [18, 20, 21], we identified the following variables as risk factors of novel coronavirus infection: baseline indices, including sex (male or female), age, body mass index (BMI), occupation (office employee, craftsperson, service employee, unemployed or retired person, healthcare staff, student or others), region (Macao native, Chinese mainland or others), personal habits (smoking, drinking, physical exercise frequency and diet preference), as well as inoculation status, such as vaccination history (unvaccinated, partially vaccinated or fully vaccinated), vaccine product types and duration from last inoculation; Additionally, the medication data, including the dose and administered frequency of Chinese proprietary and medicating duration were also collected. Apart from these, series of outcomes were reported, such as the changes of COVID-19 induced symptoms (sore throat, cough, dry mouth or throat, fever, running nose, yellow phlegm, white phlegm, myalgia, fatigue, and so on), duration of quarantine and nadir cycle threshold (Ct) value.

### Statistical analysis

According to the normality of distribution, evaluated by Kolmogorov–Smirnov, data were described with mean ± standard deviation (SD) or median (inter-quartile range (IQR)). Classified variables were described with frequency (ratio) and analyzed by chi-squared test of binary table or multi-contingency table. Data failed to meet the chi-squared test conditions were examined using chi-squared test with continuity correction. We partitioned the dose of medicine into intervals according to an equal percentile approach, ultimately reaching a discretization of the independent variable in order to obtain a more stable fitting model (low, middle and high doses), as well as a more intuitive display of a dose–response-like relationship between the independent variable and dependent variable. Binary logistic regression and Cox regression were applied to evaluate the relationship between drug using status and Ct value change, as well as vaccination status and duration of Ct value turning negative, respectively. Briefly, the positive Ct value was assigned with a value of 1, while the negative one was assigned with a value of 0. Population baseline characteristics were included as covariates, based on biological and statistical considerations, which covered sex, age, BMI, occupation, region, personal habits and inoculation status in the adjusted model. Additionally, no collinearity was found in all the covariates via collinearity diagnosis. As to Cox regression model, we set the positive or negative Ct value when discharging as the outcome variable, along with the quarantine duration as survival time. The type of vaccine products was considered as the multi-stratified covariate, which aimed to explore the effect of different kinds of vaccines on the rate of Ct value turning negative during quarantine. Data were analyzed using IBM SPSS (Chicago, Illinois, USA) and *p*-value < 0.05 was considered significant statistically.

## Results

### Baseline information of participants

**Fig. 1:**
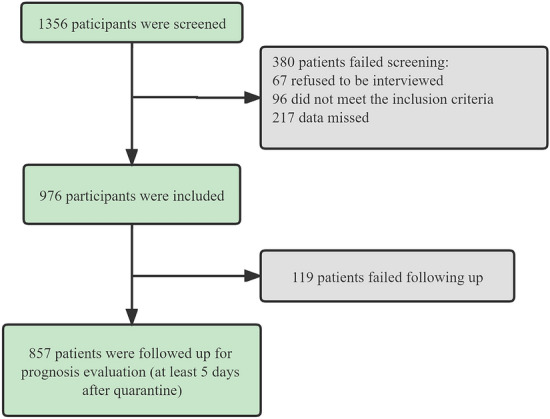
Flow chart of the population recruiting process

A total of 976 subjects quarantined in Macao hotels were included according to the eligibility criteria, and 857 patients participated the follow-up interview (Fig. 1). As displayed in Table 1, more than half of the participants were female (508, 52.0%), Macao native (643, 65.8%) and preferred stimulating diet (581, 59.5%), such as fried food, spicy dishes or strong tea. Additionally, among the registered patients, engaged in the service industry accounted for a higher proportion (service employee, 311, 31.8%). In terms of personal habits, more subjects never took physical exercises (328, 33.6%). We also found that most participants had received at least one dose of vaccine (partially vaccinated, 421, 43.1%; fully vaccinated, 434, 44.4%), of those who were inoculated, most received the authorized type of COVID-19 inactivated vaccine produced by China National Biotech Group (CNBG, 729, 74.6%). During the quarantine period, majority of patients were treated with LHQW capsules (899, 92.1%) for approximately one to two weeks (1–6 days, 378, 38.7%; 7–12 days, 367, 37.6%).

**Table 1:** Baseline demographic information and drug use or vaccination characteristics

Characteristics	Population
No. of participants (n (%))	976
Gender (n (%))	
Male	468 (47.9%)
Female	508 (52.0%)
Age (year, mean ± SD)	38.34 ± 15.63
BMI (kg/m^2^, median (IQR))	22.4 (20.0, 24.9)
Occupation (n (%))	
Office employee	152 (15.5%)
Craftsperson	202 (20.2%)
Service employee	311 (31.8%)
Healthcare staff	124 (12.7%)
Unemployed or retired person	20 (2.0%)
Student	111 (11.3%)
Others	56 (5.7%)
Region (n [%])	
Macao (China) native	643 (65.8%)
Chinese mainland	150 (15.3%)
Others	183 (18.7%)
Smoker {[n (%)]}	182 (18.6%)
Alcohol consumer {[n (%)]}	332 (34.0%)
Physical exercise [n (%)]	
Never	328 (33.6%)
Seldom	230 (23.5%)
< 3 times per week	194 (19.8%)
≥ 3 times per week	213 (21.8%)
Stimulating diet preference [n (%)]	581 (59.5%)
Vaccination history [n (%)]	
Unvaccinated	121 (12.3%)
Partially vaccinated	421 (43.1%)
Fully vaccinated	434 (44.4%)
No. of days from last time (median (IQR))	141.0 (89.0, 230.0)
Vaccine product [n (%)]	
CNBG	729 (74.6%)
mRNA	82 (8.4%)
CNBG double times and mRNA	42 (4.3%)
mRNA double times and CNBG	2 (0.2%)
Medical treatment (frequency, [n (%)]	
Lianhua Qingwen capsule	899 (92.1%)
Huoxiang Zhengqi capsule	214 (21.9%)
Duration of medicating [n (%)]	
Forget	26 (2.6%)
0	21 (2.1%)
1–6 days	378 (38.7%)
7–12 days	367 (37.6%)
> 12 days	184 (18.8%)

### Outcomes of patients with COVID-19 after quarantine

**Fig. 2:**
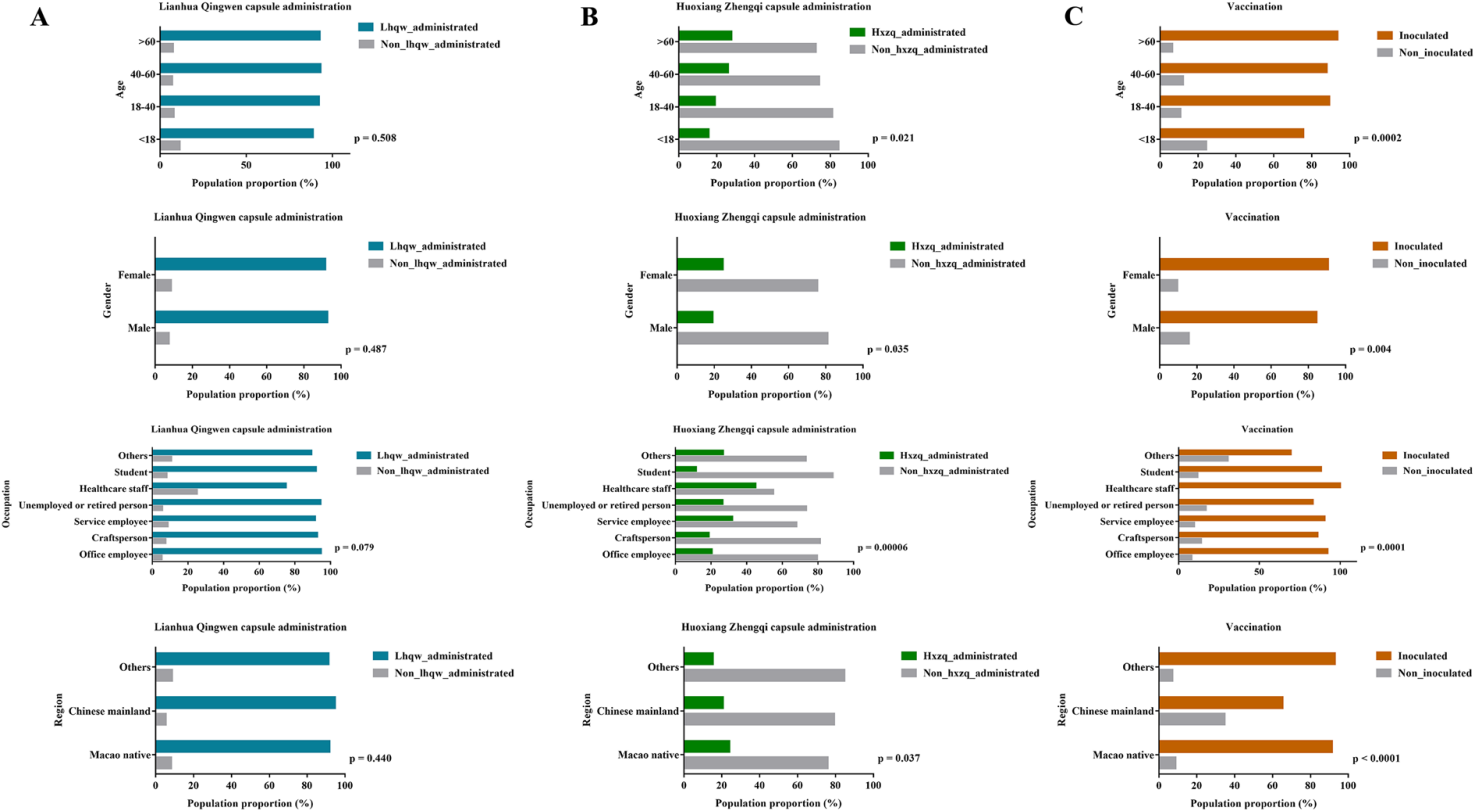
Tendency to different medicines options and inoculation status according to various stratification

**Fig. 3:**
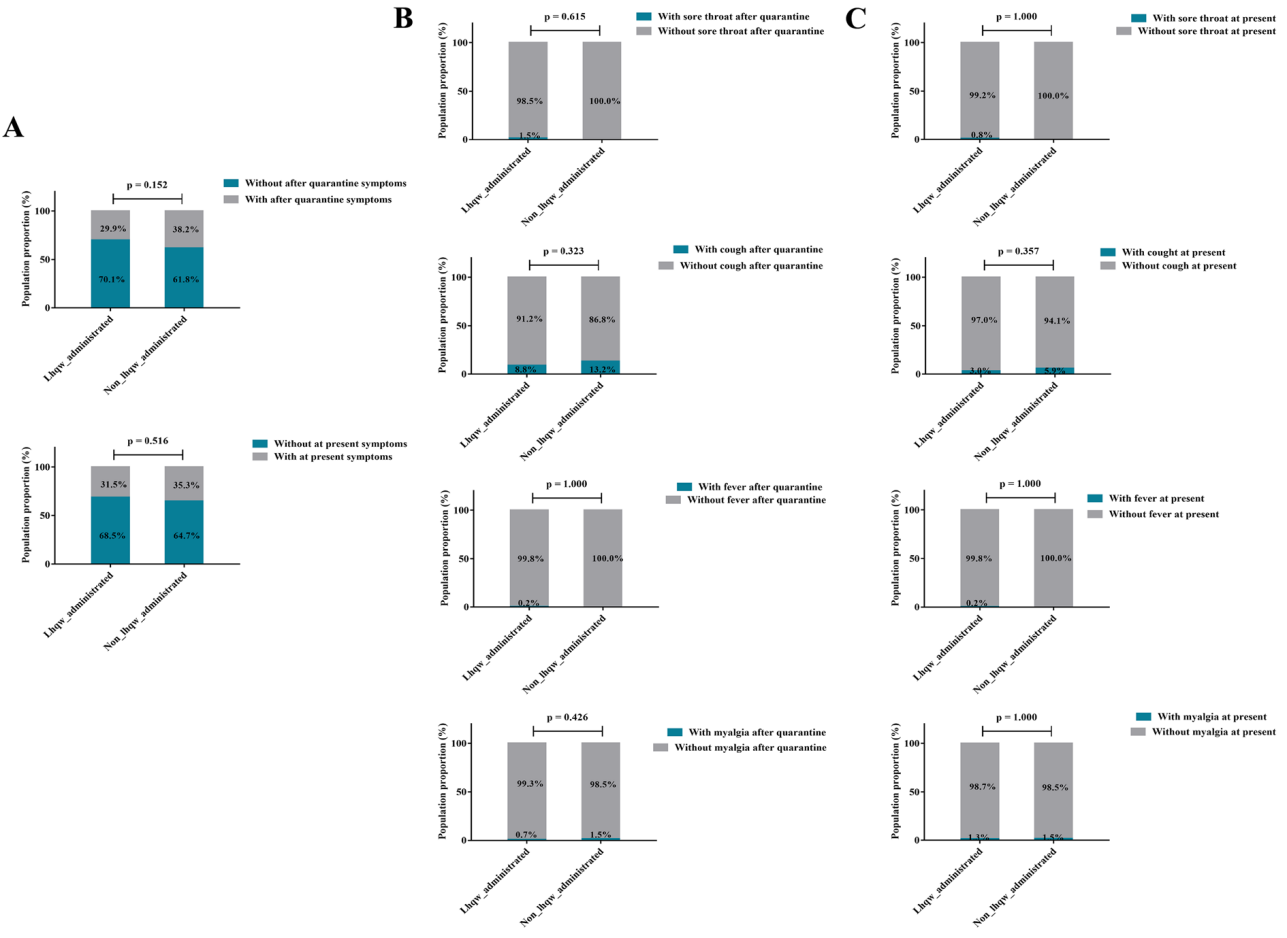
Symptomatic differences in administration of LHQW

The major symptoms induced by COVID-19 were exhibited in Table 2, among which, sore throat (384, 40.2%), cough (547, 57.3%) and fever (659, 69.0%) were the most common ones of patients at the very beginning of quarantine. However, after proper Chinese proprietary medicine therapy, the occurrence of symptoms was significantly decreased, coupled with evidently increased amount of negative Ct value. In addition, we found that the duration of quarantine mainly distributed within one month (median (IQR), 21.0 (18.0, 25.0)). Subsequently, the medicine option was displayed according to the stratification of demographic indices (Fig. 2A–B). The employment of Chinese proprietary medicine and vaccination status were different in various age, sex, occupation and region, for example, patients aged over 60 years old were prone to take HXZQ capsules and inoculated (age > 60 years old, ratio of taking HXZQ capsules = 27.7%, ratio of vaccination = 93.6%), so did the female group (female, ratio of taking HXZQ capsules = 24.6%, ratio of vaccination = 90.6%). Participants engaged in healthcare industry were more likely to accept HXZQ treatment and get vaccinated (healthcare staff, ratio of taking HXZQ capsules = 45.0%, ratio of vaccination = 100%), while patients from Macao native were more active in receiving HXZQ medication (Macao native, ratio of taking HXZQ capsules = 24.1%) (Fig. 2A–C). In addition, with or without LHQW capsule administration, there was no significant effect on the following indices, including the ratio of presence or absence of COVID-19 related symptoms, as well as the prognosis symptoms (Fig. 3A). Also, according to the indications for LHQW capsule, we found that whether or not this drug was taken, had no influence on the post-treatment and prognostic changes in the proportion of sore throat, cough, fever, and myalgia symptoms in patients with positive symptoms before quarantine (Fig. 3B–C).

**Table 2:** Symptoms and CT value alteration during quarantine

Variables	Initial occurrence (n [% of participants])	After quarantine occurrence (n [% of participants])	χ^2^	*p* value
Symptom				
Sore throat	384 (40.2%)	12 (1.3%)	440.9	** < 0.0001**
Cough	547 (57.3%)	83 (8.7%)	509.9	** < 0.0001**
Dry mouth or throat	228 (23.9%)	30 (3.1%)	175.7	** < 0.0001**
Fever	659 (69.0%)	3 (0.3%)	994.9	** < 0.0001**
Running nose	218 (22.8%)	10 (1.0%)	215.5	** < 0.0001**
Yellow phlegm	148 (15.5%)	3 (0.3%)	151.2	** < 0.0001**
White phlegm	145 (15.2%)	27 (2.8%)	89.0	** < 0.0001**
Myalgia	226 (23.7%)	8 (0.8%)	231.5	** < 0.0001**
Fatigue	213 (22.3%)	43 (4.5%)	130.4	** < 0.0001**
Diarrhea	174 (18.2%)	15 (1.6%)	148.5	** < 0.0001**
Constipation	41 (4.3%)	7 (0.7%)	24.7	** < 0.0001**
Chest tightness	52 (5.4%)	15 (1.6%)	21.2	** < 0.0001**
Shortness of breath	59 (6.2%)	28 (2.9%)	11.6	** < 0.001**
Palpitation	32 (3.4%)	5 (0.5%)	20.1	** < 0.0001**
Insomnia	93 (9.7%)	23 (2.4%)	45.0	** < 0.0001**
Nausea or vomit	37 (3.9%)	1 (0.1%)	34.8	** < 0.0001**
Dizziness	102 (10.7%)	10 (1.0%)	80.3	** < 0.0001**
Headache	260 (27.2%)	15 (1.6%)	255.0	** < 0.0001**
Abdominal pain	31 (3.2%)	4 (0.4%)	21.2	** < 0.0001**
Change sense of smell and taste	93 (9.7%)	14 (1.5%)	61.8	** < 0.0001**
Duration of quarantine (median (IQR))	21.0 (18.0, 25.0)			
Negative CT value	29 (2.9%)	595 (60.9%)	774.6	** < 0.0001**

The bold values pointed to statistical significance

### Correlation of Chinese proprietary medicine therapy and CT value alteration

Next, the dosage and duration of medicating were stratified into low, middle, and high levels (or low, high levels) according to data distribution and were further linked with the Ct value (after quarantine) in order to establish the binary logistic regression model. As displayed in Table 3, the demographic indices (age, sex, BMI, occupation, region), personal habits (smoking, drinking, physical exercises, stimulating diet preference) and vaccination status (dose of vaccine, type of vaccine product, days from last dose) were taken account in the adjusted model compulsively, due to their underlying relationship with the medication and COVID-19 PCR results. Meanwhile, the crude model was established via directly incorporating the independent variable without any correction. We found that middle dose (4–5 boxes) of LHQW was negatively associated with positive Ct value (crude, − 0.32 ± 0.16, *p* = 0.05; adjusted, − 0.37 ± 0.19, *p* = 0.04), which indicated the protective effect of this Chinese proprietary medicine on favorable patients outcome after quarantine. However, the high level of treating duration exhibited evidently positive association with positive Ct value, suggesting that the longer medication time may be disadvantageous to the Ct value turning negative.

**Table 3:** Association between drug use and CT value alteration

Variables		CT value alteration (n = 883)			
		Crude		Adjusted^a^	
		Linear coefficient ± S.E	*p* value	Linear coefficient ± S.E	*p* value
Dose of Lianhua Qingwen capsule	Low	0		0	
	Middle	− 0.32 ± 0.16	**0.05**	− 0.37 ± 0.19	**0.04**
	High	0.12 ± 0.63	0.85	0.16 ± 0.65	0.81
Dose of Huoxiang Zhengqi capsule	Low	0		0	
	High	− 0.25 ± 0.18	0.17	− 0.37 ± 0.21	0.08
Duration of medicating	Low	0		0	
	Middle	0.17 ± 0.17	0.31	0.24 ± 0.19	0.21
	High	0.40 ± 0.21	0.06	0.55 ± 0.24	**0.02**

The bold values pointed to statistical significance

^a^Model was adjusted for demographic indices, personal habits and vaccination information (vaccination history, vaccine product and No. of days from last time)

### Effect of vaccination on CT value change

**Fig. 4:**
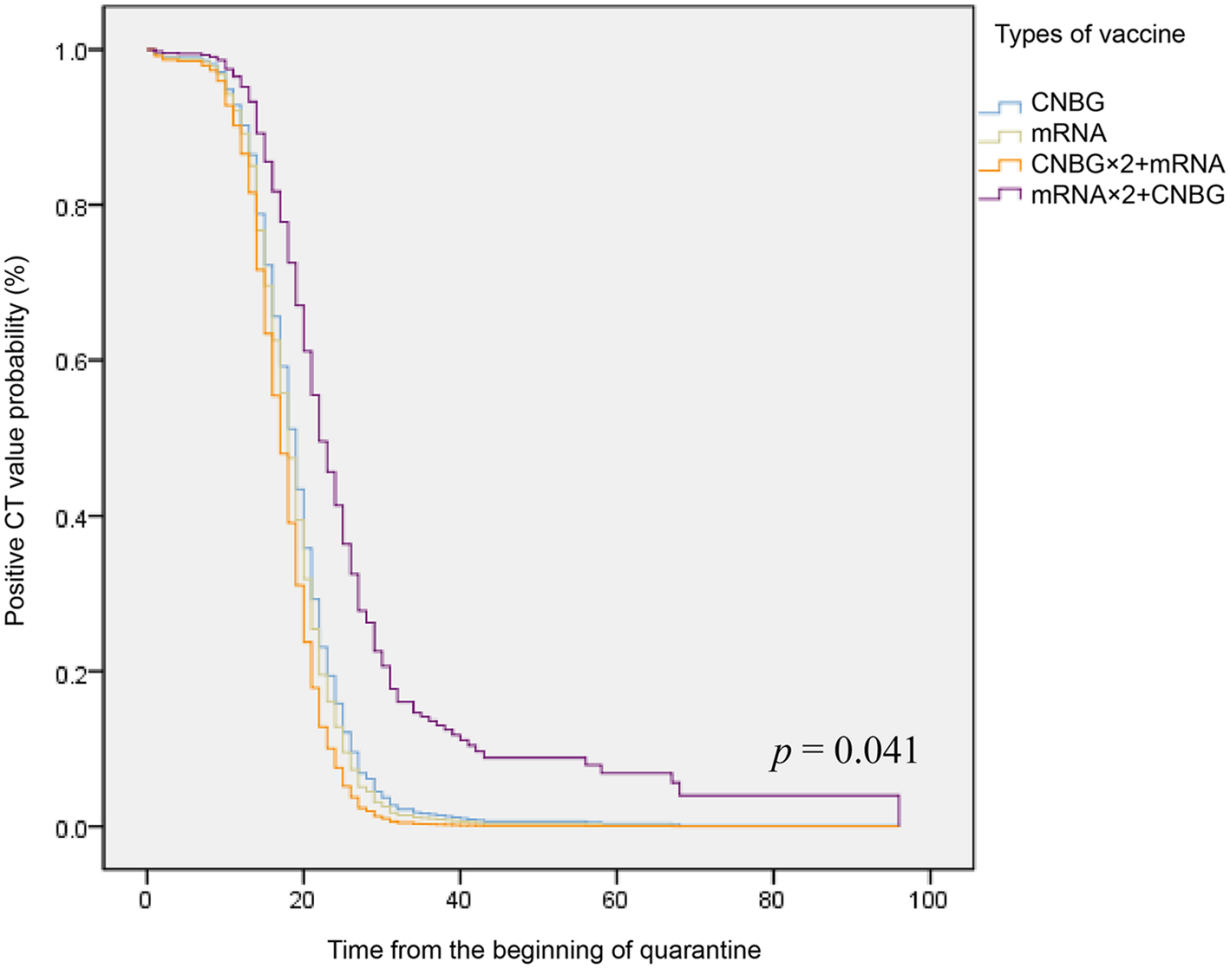
The Cox regression model for the promoting factors of Ct value turning negative rate

In order to identify the promoting factors of Ct value turning negative rate, we employed the Cox regression model. As shown in Fig. 4, compared with the group that only used CNBG inactivated vaccine, the group of two doses of CNBG and one dose of mRNA played a more active role in reducing positive Ct value possibility (HR = 1.40, 95% CI (1.01, 1.94)), however, the only use of mRNA and two doses of mRNA with one dose of CNBG did not show statistically significance (mRNA, HR = 1.11, 95% CI (0.88, 1.42); mRNA × 2 + CNBG, HR = 0.48, 95% CI (0.12, 1.93)). Moreover, all demographic baseline information did not display any significant correlation with Ct turning negative time (Additional file 1: Table S1), which suggested that they were not the independent risk factor of Ct value altering rate.

### Factors evaluation of prognosis symptoms

With the normalization of the COVID-19 pandemic, the prognostic problem after virus infection has become a great concern in public society. Here, after five days to even two months of follow-up, the prognosis symptoms were collected and the feasible factors, such as symptoms at the beginning of quarantine, self-feeling of medication, were statistically analyzed through logistic regression (Table 4).

**Table 4:** Correlation between featured variables and prognosis symptoms

Variables	Prognosis symptoms (n = 976)			
	Crude		Adjusted^a^	
	Linear coefficient ± S.E	*p* value	Linear coefficient ± S.E	*p* value
Initial symptoms	0.90 ± 0.51	0.08	0.80 ± 0.52	0.12
After quarantine symptoms	1.49 ± 0.18	** < 0.0001**	1.38 ± 0.18	** < 0.0001**
Subjective medicating evaluation	− 0.11 ± 0.09	0.25	− 0.08 ± 0.10	0.39
Times of vaccine	− 0.08 ± 0.17	0.64	0.10 ± 0.19	0.59
No. of days from last dose	− 0.002 ± 0.001	0.08	− 0.001 ± 0.001	0.49
Type of vaccine	0.10 ± 0.15	0.50	0.06 ± 0.16	0.71

The bold values pointed to statistical significance

^a^Model adjusted for demographic indices and personal habits (smoker, alcohol consumer, physical exercises and stimulating diet preference)

In terms of risk factor, symptoms after quarantine were positively associated with the follow-up ones (adjusted, B ± SEM = 1.38 ± 0.18, *p* < 0.0001), that is, the more obvious the symptoms were at the beginning of quarantine, the less optimistic the prognosis was. Nevertheless, patients’ subjective evaluation of medicating was negatively related to prognostic symptoms, though not significant (adjusted, B ± SEM = − 0.08 ± 0.10, *p* = 0.39). In addition, the number of days since the last dose of vaccine was also slightly negatively associated with patients recovery (adjusted, B ± SEM = − 0.001 ± 0.001, *p* = 0.49).

## Discussion

The present study, to our knowledge, for the first time deciphered the Chinese proprietary medicine and vaccination effects on indices related to COVID-19 and prognosis from the lens of statistical analysis in Macao. Here, we observed that over half of patients were female, of which, people engaged in service industry, such as tourism, gambling, retail, and catering, accounted for a high proportion. These findings were consistent with current reports in Hong Kong [22] and Egypt [23]. The proportion of service employee in quarantined patients may be derived from the local industrial structure, as the tertiary industry is the local pillar industry [24]. In terms of personal habits, more of the quarantine subjects were those who never took physical exercises. This was quite reasonable as a spectrum of epidemiological studies had declared that regular physical activity was associated with a lower risk of COVID-19 infection [25, 26], moreover, the cardiopulmonary benefits brought by exercise could reduce the possibility of being hospitalized due to COVID-19 [27, 28]. Meanwhile, nutrients in the proper and healthy diet could effectively reduce oxidative stress and inflammation, which particularly vital during the COVID-19 crisis [29]. Conversely, irritating diet, such as fried food, has been demonstrated to increase the systemic inflammation [30] which is very disadvantageous to coronavirus struggle [29, 31]. In our study, we found that nearly 60% of participants preferred irritating food (581, 59.5%), among which, fried food and strong coffee were favored by more patients than spicy food and strong tea (fried food preference, 44.8%; strong coffee, 52.0%, Additional file 1: Table S2).

In addition, at the beginning of quarantine, over half of the patients showed fever and cough. However, after quarantine and medication, these systemic and upper respiratory symptoms were evidently ameliorated. We also observed that middle dose of LHQW had the significant protective efficacy in promoting Ct value turning negative. In fact, research on LHQW, HXZQ and western medicine showed that the combination treatment of these drugs against COVID-19 had the potential of reducing the rate of progression to severe cases and improve patient prognosis [32].Specifically, as the symptomatic treatment from Chinese proprietary medicine, LHQW has been recommended to treat mild or moderate COVID-19 by the Evidence-Based Medicine Chapter of the China International Exchange and Promotive Association for Medical and Health Care (CPAM) and the Chinese Research Hospital Association (CRHA) [33, 34]. Two population studies on LHQW combined with regular treatment showed that patients with COVID-19 suffered less from fever, cough, and weakness [35, 36], which were different from our findings. We suspected that it may result from the too-small sample size of patients who did not take LHQW capsules (894 patients with symptoms at the beginning, 7.6% of them not LHQW administrated) and we did not control the independent variable intentionally, making the causality result on drug using leading to symptoms alteration weak. However, the effect of LHQW on patients’ Ct value turning negative did help us find the potential of this drug on COVID-19 treatment, which aligned to the positive report of LHQW functioning on anti-virus replication of SARS-CoV-2 [37]. Additionally, pertaining to the target prediction of LHQW, research displayed that the highly enriched targets were related to inflammation and oxidative stress response, such as the interleukin-17 (IL-17) pathway and the nuclear factor kappa-B (NF-κB) pathway [38]. The component study on LHQW also showed that bioactive ingredients such as quercetin, naringenin, β-sitosterol, luteolin, and stigmasterol may target on regulating androgen receptor (AR), myeloperoxidase (MPO), epidermal growth factor receptor (EGFR), insulin and aryl hydrocarbon receptor (AHR) gene expression against COVID-19 [39]. Taken together, our study confirmed the potential efficacy of the Chinese proprietary medicine through the self-control of patients, nevertheless, the possibility of self-recovery could not be ruled out in the present analysis, and therefore brought a challenging causality between drug treatment and Ct value alteration, which is the limitation in the study due to the lack of control group. Hence, the present study suggested the potential positive association between the Chinese proprietary medication and COVID-19 related index, but not the direct causal relationship of these two variables.

Vaccination has been the focal point since the pandemic onset. Most of the authorized vaccine performed well in the prevention of severe cases of COVID-19 [40]. In our study, patients with certain characteristics, especially engaged in healthcare and over 60 years old, were prone to receive vaccination. Being at high risk of exposure to virus [41], 28.99% of medical staff was reported to be infected in the very first COVID-19 pandemic of Zhongnan Hospital Affiliated to Wuhan University. Meanwhile, according to the related report from WHO, by April 2020, a total of 22,703 COVID-19 cases have been documented over 52 countries worldwide. However, under the heavy burden of epidemic [42], there still remained high level of vaccine hesitancy among healthcare staff in some countries [43, 44], on the contrary, our study found the inclination of vaccination in medical staff, which may be owing to good publicity and authentic information on the role of inoculation [43]. Moreover, during the Omicron wave in Shanghai since March 2022, older age has been demonstrated as the risk factor of severe or critical case, and among which, fully vaccinated shortened the viral shedding time, thus evidently protect patients from severe infections [21], meanwhile, multiple times of vaccination were able to further prevent symptomatic diseases, and might play a protective role in the resistance of future variants [45]. By far, three years on from the administration of the first experimental COVID-19 vaccine doses to human in March 2020, and 18 vaccines have been designated for employment [40]. There are several main types of vaccine platforms, including mRNA, inactivated virus, viral vector and protein subunit [46–49]. In the present study, patients inoculated with inactivated virus vaccine (CNBG) and mRNA vaccine accounted for the highest proportion. We also found that two times of CNBG, coupled with one time of mRNA exhibited a better efficiency in reducing Ct positive rate during 20 days of quarantine. In fact, this kind of mixed inoculation is now very common all over the world. Study suggested the population vaccinated with Oxford-AstraZeneca (viral vector vaccine) the first time and Pfizer-BioNTech (mRNA vaccine) the second time triggered 11.5 folds in higher anti-spike response of immunoglobulin G (IgG) and IgA than those received both times of AstraZeneca [50]. Similar reports from Britain [51] and Spain [52] have affirmed the value of mixed inoculation.

With the continuous liberalization of prevention policy on COVID-19, the normalization of the pandemic has been infiltrated into various areas in China. As a result, the prognosis of infection has become a great concern in the whole society. Adverse prognostic events were often related to severe cases [53]. Recent evidence revealed that cardiovascular involvement and inflammation were highly associated with poor prognosis and mortality [54–58]. In addition, neurological symptoms, such as headache, fatigue, and loss of olfactory sensation, as well as gastrointestinal reactions, were common adverse prognostic events [59, 60]. In the present study, we found that symptoms after quarantine were positively associated with prognosis, rather than initial symptoms. Intriguingly, patients’ subjective evaluation about medication displayed negative correlation with prognosis symptoms, meaning more positive self-evaluation, less prognostic symptoms, which was similar to one health quality study on patients with intensive care unit admission. This study illustrated that health perception scores at 2 months after admission was positively associated with that at 12 months after admission [61].

As to the limitation of the present study, there were many covariates that could have influenced the progression of COVID-19 as well as medication or vaccination which were unmeasured here, such as the underlying disease of patients, especially cardiopulmonary involvement, the corresponding indices like clinical lung function tests, chest Ct scanning were lack [62, 63]. Patients with underlying medical conditions, especially those with longstanding infections, chronic metabolism disorder, or cancer, are more likely to suffer from more severe symptoms, thus leading to ineffective medication, or longer medicating duration and higher drug doses. Other covariates like education and economic levels, are also engaged in obscuring the association of drug administration and COVID-19 progression [64], as patients with higher education and economic level may more acknowledge the significance of instant quarantine and positive medicating, and hence, more easily being enrolled in our survey and resulting in the occurrence of bias. We admitted the weakness inherited in retrospective study, including the difficulty of re-collecting specific information, such as the previously unmeasured confounders, like the prevalence of underlying diseases and economic income levels. Most notably, due to the lack of human control of the independent variable, this type of study cannot directly determine the causal relationship between the independent variable and the dependent variable but can only provide clues to correlations. As for the limitation of self-control, we realize that it may introduce bias in the selection of the study population. For example, the patients included in the study were mainly Macao native, who, due to the high population density and mobility of people in the area, may have a higher probability of being exposed to viral strains, which may affect the accuracy of the conclusions we draw, and therefore the conclusions we obtain are cautious.

Taken together, the present study manifested that the mixed vaccination provided the possibility to accelerate the discharge of COVID-19 patients, and the treatment of Chinese proprietary medicine brought new opportunities for remission of the disease, which implied that mixed inoculation and necessary medication remain pivotal tools for prevention and palliation during epidemic, and importantly, whether current strains will mutate and local epidemics will change remains to be seen, hence, more effective vaccines and drugs are still needed, and more rigorous, large-sample, controlled-variable, population-based prospective studies of these prophylactic and therapeutic approaches should be conducted [65, 66]. Moreover, considering the difference of demographic characteristics of Macao, compared with Chinese mainland, such as the higher population density and mobility, site-specific prevention and control strategies should also be adapted over time as this novel evidence emerges in the future.

## Conclusion

Our retrospective study, for the first time, delineated the protective effects of LHQW capsule and mixed vaccine on patients quarantined in Macao, meanwhile, we identified certain risk factor of prognosis from the lens of statistical data. These provide novel clues for the prevention and medication of COVID-19 locally in the future, and of note, as the normalization of COVID-19 pandemic, more methods, and strategies for improving the prognosis of COVID-19 can be put forward through the present evidence.

### Supplementary Information

**Additional file 1.** **Table S1**: Correlation between baseline characteristics and CT value alteration. **Table S2**: Dietary preference in stimulating food of quarantined participants.

## Data Availability

The data used and/or analyzed during the present study are available from the corresponding author on reasonable request.

## Abbreviations

WHO: World Health Organization
LHQW: Lianhua Qingwen
HXZQ: Huoxiang Zhengqi
TCM: Traditional Chinese Medicine
BMI: Body mass index
CI: Confidence interval
Ct: Cycle threshold
CNBG: China National Biotech Group
