# Supplementary material for: The effects of Chinese proprietary medicine and vaccination on patients with COVID-19: a retrospective study in Macao
Source: Chin Med. 2024 Jan 23;19:15. doi: 10.1186/s13020-023-00877-8 (PMC10807168; doi:10.1186/s13020-023-00877-8)
Supplement: Supplementary file 1 — Additional file 1. Table S1: Correlation between baseline characteristics and CT value alteration. Table S2: Dietary preference in stimulating food of quarantined participants. [file 13020_2023_877_MOESM1_ESM.docx]

**Table S1 Correlation between baseline characteristics and Ct value alteration**

| Characteristics | | OR and 95% CI | | | B | SE | p value |
| --- | --- | --- | --- | --- | --- | --- | --- |
|  |  | OR | lower limit | upper limit |  |  |  |
| Gender a |  | 1.41 | 0.97 | 2.04 | 0.34 | 0.19 | 0.07 |
| Age |  | 0.99 | 0.97 | 1.00 | -0.01 | 0.01 | 0.14 |
| BMI |  | 1.03 | 1.00 | 1.06 | 0.03 | 0.02 | 0.10 |
| Occupation b | Craftsperson | 0.57 | 0.31 | 1.03 | -0.57 | 0.31 | 0.06 |
|  | Service employee | 0.65 | 0.41 | 1.04 | -0.43 | 0.24 | 0.07 |
|  | Healthcare staff | 0.70 | 0.38 | 1.31 | -0.36 | 0.32 | 0.26 |
|  | Unemployed or retired person | 0.78 | 0.26 | 2.37 | -0.25 | 0.57 | 0.66 |
|  | Student | 0.50 | 0.24 | 1.05 | -0.69 | 0.38 | 0.07 |
|  | Others | 0.77 | 0.34 | 1.73 | -0.27 | 0.42 | 0.53 |
| Region c | Chinese mainland | 0.79 | 0.45 | 1.39 | -0.24 | 0.29 | 0.41 |
|  | Others | 1.06 | 0.63 | 1.79 | 0.06 | 0.27 | 0.83 |
| Smoking d |  | 0.74 | 0.46 | 1.19 | -0.31 | 0.25 | 0.22 |
| Drinking e |  | 1.16 | 0.79 | 1.71 | 0.15 | 0.20 | 0.45 |
| Physical exercise f | Seldom | 0.96 | 0.63 | 1.47 | -0.04 | 0.22 | 0.86 |
|  | < 3 times per week | 1.08 | 0.68 | 1.69 | 0.07 | 0.23 | 0.76 |
|  | ≥ 3 times per week | 0.92 | 0.59 | 1.42 | -0.09 | 0.22 | 0.70 |
| Stimulating diet preference g |  | 0.95 | 0.68 | 1.34 | -0.05 | 0.17 | 0.79 |

a: compared with male gender;

b: compared with office employee;

c: compared with Macao native;

d: compared with participants who don't smoke;

e: compared with participants who don't drink;

f: compared with participants who never exercise;

g: compared with participants who don't prefer stimulating diet.

**Table S2 Dietary preference in stimulating food of quarantined participants**

| Dietary preference (n=581) | Blank | Dislike | Ordinary | Preferred | Much preferred | Preference |
| --- | --- | --- | --- | --- | --- | --- |
| Fried food | 2 (0.3%) | 87 (14.9%) | 232 (39.9%) | 221 (38.0%) | 39 (6.7%) | 260 (44.8%) |
| Spicy food | 5 (0.8%) | 126 (21.6%) | 188 (32.3%) | 195 (33.5%) | 67 (11.5%) | 262 (45.1%) |
| Strong tea | 1 (0.1%) | 189 (32.5%) | 184 (31.6%) | 152 (26.1%) | 55 (9.4%) | 207 (35.6%) |
| Strong coffee | 0 | 171 (29.4%) | 108 (18.5%) | 210 (36.1%) | 92 (15.8%) | 302 (52.0%) |
